# The Influence of Maternal Lifestyle Factors on Human Breast Milk Microbial Composition: A Narrative Review

**DOI:** 10.3390/biomedicines12112423

**Published:** 2024-10-22

**Authors:** Irene Bianco, Chiara Ferrara, Francesca Romano, Federica Loperfido, Francesca Sottotetti, Dana El Masri, Alessandra Vincenti, Hellas Cena, Rachele De Giuseppe

**Affiliations:** 1 Laboratory of Dietetics and Clinical Nutrition, Department of Public Health, Experimental and Forensic Medicine, University of Pavia, 27100 Pavia, Italy; irene.bianco@unipv.it (I.B.); chiara.ferrara01@unipv.it (C.F.); romanofrancesca93@gmail.com (F.R.); federica.loperfido@unipv.it (F.L.); dana.elmasri01@universitadipavia.it (D.E.M.); alessandra.vincenti@unipv.it (A.V.); hellas.cena@unipv.it (H.C.); rachele.degiuseppe@unipv.it (R.D.G.); 2 Clinical Nutrition Unit, General Medicine, Istituti Clinici Scientifici (ICS) Maugeri, Istituto di Ricovero e Cura a Carattere Scientifico (IRCCS), 27100 Pavia, Italy

**Keywords:** human breast milk, microbiota, maternal dietary pattern, lifestyle, breastfeeding

## Abstract

Human breast milk (HBM) is considered the gold standard for infant nutrition due to its optimal nutrient profile and complex composition of cellular and non-cellular components. Breastfeeding positively influences the newborn’s gut microbiota and health, reducing the risk of conditions like gastrointestinal infections and chronic diseases (e.g., allergies, asthma, diabetes, and obesity). Research has revealed that HBM contains beneficial microbes that aid gut microbiota maturation through mechanisms like antimicrobial production and pathogen exclusion. The HBM microbiota composition can be affected by several factors, including gestational age, delivery mode, medical treatments, lactation stage, as well as maternal lifestyle habits (e.g., diet, physical activity, sleep quality, smoking, alcohol consumption, stress level). Particularly, lifestyle factors can play a significant role in shaping the HBM microbiota by directly modulating the microbial composition or influencing the maternal gut microbiota and influencing the HBM microbes through the enteromammary pathway. This narrative review of current findings summarized how maternal lifestyle influences HBM microbiota. While the influence of maternal diet on HBM microbiota is well-documented, indicating that dietary patterns, especially those rich in plant-based proteins and complex carbohydrates, can positively influence HBM microbiota, the impact of other lifestyle factors is poorly investigated. Maintaining a healthy lifestyle during pregnancy and breastfeeding is crucial for the health of both mother and baby. Understanding how maternal lifestyle factors influence microbial colonization of HBM, along with their interactions and impact, is key to developing new strategies that support the beneficial maturation of the infant’s gut microbiota.

## 1. Introduction

Human breast milk (HBM) is widely regarded as the gold standard of infant nutrition, offering an unparalleled source of essential nutrients tailored specifically for the newborn during the crucial neonatal period [1]. This remarkable fluid is highly complex, comprising diverse cellular components and bioactive molecules, all working together to support the infant’s growth, immune development, and overall health [2]. “Human milk” refers to milk produced by lactating parents and includes (i) breast milk produced by a parent that is given directly to infants through the breast or expressed by the breastfeeding parent and then given to the infant; (ii) donor milk produced by breastfeeding individuals is stored and then donated (e.g., human milk banks) or given to infants other than their children [3]. Breastfeeding is one of the exposure factors that can modify the composition of the newborn’s gut microbiota and, therefore, perpetuate the state of newborn health [4]. Notably, breastfed children exhibit a decreased risk of developing diseases in their lifespan, such as gastrointestinal infection, acute otitis media, and infectious diarrhea, compared to infants fed with formula milk [5,6].

As widely described, HBM is recognized as one of the most important postpartum elements modulating metabolic and immunologic programming related to the child’s health [7,8]; this is partly due to the presence of a specific HBM microbiota, a source of beneficial, non-pathogenic, and probiotic bacteria that may positively affect the infant gut microbiota maturation [9]. Several studies have also shown that breastfed babies had a lower risk of chronic diseases such as allergies, asthma, diabetes, and obesity in childhood and adulthood [10,11,12]. All of this could be related to the composition of the HBM microbiota and its multiple mechanisms [13], including the production of antimicrobial compounds against pathogenic bacteria, the exclusion of competitors, the prevention of adherence of pathogenic bacteria to the intestinal epithelium, and the increase in intestinal mucosal production [14].

Alterations in the composition of the HBM microbiota can be ascribed to several exposure factors, such as maternal health (presence of certain pathologies including obesity, allergies, celiac disease, mastitis) [15,16,17], maternal dietary habits and lifestyle factors [18], gestational age (term vs. preterm birth) [19], delivery mode (vaginal birth vs. C-section birth) [7], medical treatment and antibiotic exposure [7], lactation period (colostrum vs. transitional milk vs. mature milk) [20]. Furthermore, it is widely believed that the bacteria colonizing HBM originate from the mother’s skin and the newborn’s oral cavity, with the infant being exposed to the mother’s vaginal and intestinal microbiota during childbirth [9].

Particularly, among these factors, it is important to highlight the crucial role that the adoption of adequate lifestyle plays in periconception and prenatal and postnatal care in maintaining general health and preventing lifestyle-related diseases, including non-communicable diseases (NCDs), throughout the entire life course [21].

Given the growing interest in identifying modifiable maternal factors that may influence the HBM microbiota, to the best of our knowledge, the present narrative review is the first to provide an overview of maternal lifestyle elements, including dietary habits, physical activity, sleep quality, smoking, and stress levels, that are associated with shaping the HBM microbiota. Special attention is given to maternal diet during lactation and its potential impact on the infant’s health. By understanding the effect of lifestyle on the formation of the HBM microbiota, it is possible to generate personalized advice to adapt to the maternal profile and encourage the adoption of lifestyle changes that can influence the composition of the newborn’s intestinal microbiota and preserve its health.

## 2. The Human Breast Milk Composition

The HBM is a dynamic, bioactive fluid that changes in composition from colostrum to late lactation and varies within feeds, diurnally, and between mothers [2]. It consists of cellular and non-cellular components coming from different sources (i.e., lactocytes) [2].

### 2.1. Non-Cellular Components of the Human Breast Milk

The non-cellular components of HBM encompass macro- and micronutrients, as well as a plethora of active compounds like immunoglobulins, human milk oligosaccharides (HMOs), growth factors, cytokines, chemokines, and epithelial and immune cells, which provide nutritional, immunological, and developmental benefits to the newborn, as well as having a key role in anti-oncogenicity, neurocognitive development, cellular communication, and differentiation [9,22,23].

HBM has a unique composition that constantly changes based on neonatal needs [24]. For instance, concerning macronutrients, colostrum, which is produced in the first few days postpartum, is rich in protein (14–16 g/L), lipids (15–20 g/L), and carbohydrates (50–60 g/L), particularly HMO (20–24 g/L), and other cellular components; transitional milk, which is produced approximately 5–14 days after giving birth and is similar in composition to colostrum [2,4]; mature milk differs completely from the previous lactation stages and consists of 90% water, 3.8% fat, 1.0% protein, and 7% lactose [20]. Regarding the micronutrients present in human breast milk, they constitute the only source of nutrients for a newborn, at least for the first six months of life [2]. Although the composition of breast milk is highly conserved, the amount of nutrients can vary depending on the mother’s diet and body reserves. Since maternal dietary habits are not always optimal, the use of supplements is recommended during breastfeeding as vitamins, minerals, and trace elements are essential for infant development, including hormone synthesis, immune system function, and antioxidant activity [2]. Besides macro and micronutrient content, HBM contains human milk oligosaccharides (HMOs), complex sugars found in significant concentrations and with unique structural diversity [25]. Although they are the fourth most abundant component of human milk after water, lipids, and lactose, they provide no direct nutritional value to the newborn [25]. However, HMOs have a prebiotic effect, and through their features, they are responsible for the microbiota composition of the baby and the composition of the bacterial community of the milk itself [2,25]. Indeed, HMOs serve as a selective food source for beneficial bacteria, particularly *Bifidobacteria* and *Lactobacilli*, which have specific enzymes that can break down HMOs, allowing them to proliferate [25]. At the same time, HMOs can prevent pathogenic bacteria from binding to the intestinal lining by mimicking the receptor structures that these pathogens would normally bind to; this reduces the colonization of harmful bacteria both in the baby’s intestines and, to some extent, in the breast milk itself [25].

### 2.2. Cellular Components of the Human Breast Milk: The Microbial Composition

The HBM is not only rich in nutrients and antibodies, which play a fundamental role in the immunological development of newborns, but also includes types of cells derived from blood and breast, including immune cells, lactocytes, stem cells, and epithelial cells [2]. These cells are accompanied by the presence of some types of bacterial communities, which influence the health and development of the infant by colonizing and shaping the gut microbiota in early life [26].

In recent years, culture-independent methods, such as 16S ribosomal RNA (16S rRNA) sequencing, clone libraries, meta taxonomy, as well as next-generation sequencing technologies, have been developed, enabling accurate identification of microbial communities in HBM [27,28] and showing high inter- and intra-individual specific profiles [1].

The microbiota of breast milk comprises over 820 taxa at a species level, predominantly from the *Proteobacteria* and *Firmicutes* phyla, with *Streptococcus* and *Staphylococcus* being the most prevalent genera [1]. Studies indicate that the majority of bacterial communities in HBM are anaerobic [28]. Consequently, the HBM microbiota serves as the second source of microbes for newborns, following exposure to the birth canal during vaginal delivery [28]. Furthermore, it has been reported that by consuming approximately 800 mL of HBM per day, an infant ingests between 10^5^ and 10^7^ bacteria daily [1].

In general, the bacteriome core of HBM includes nine genera, such as *Staphylococcus*, *Streptococcus*, *Serratia*, *Pseudomonas*, *Corynebacterium*, *Ralstonia*, *Propionibacterium*, *Sphingomonas*, and *Bradyrhizobium*, representing half of the microbial milk community, although their abundance can vary across milk samples [28]. Currently, the shape of the HBM microbiota is still unclear; however, two conceivable mechanisms have been proposed to elucidate the composition of the milk microbiota [29].

First, the notion of “retrograde transfer” describes the external influx of bacteria coming from the periareolar skin and the baby’s mouth, which can also influence the composition of the infant’s gut microbiota shaping [29,30]. For instance, a study conducted by Biagi and colleagues [31] on 36 healthy mother–child pairs found several operational taxonomic units (OTUs) belonging to the genera *Bifidobacterium*, *Streptococcus*, and *Staphylococcus* in both the oral and fecal microbiota of the child as well as in the HBM microbiota. This could support the hypothesis that the baby’s mouth can play a bidirectional role: on the one hand, it could influence the composition of the newborn’s intestinal microbiota throughout swallowing; on the other hand, it could, instead, influence the colonization of the maternal milk ducts during suckling [31].

Second, the most widely accepted hypothesis, known as the “enteromammary pathway” (also known as the Gut–Breast Axis), illustrates the intricate connection between the mother’s gut health and the quality of her breast milk, emphasizing the role of lifestyle factors in shaping maternal and infant health outcomes [32,33]. In this pathway, where the translocation of internal bacteria from the mother’s gastrointestinal (GI) tract into the mammary gland, driven by immune cells during the late stages of pregnancy, the dendritic cells of the maternal intestinal mucosa engulf the mother’s intestinal bacteria and translocate through the lymphatic or circulatory system to the mammary gland [32,33]. In this manner, the maternal GI tract is a source of bacteria for the HBM microbiota, and the maternal gut microbiota is vertically transferred to the infant via HBM [34]; *Lactobacilli*, *Staphylococci*, *Enterococci*, and *Bifidobacteria* would be transferred through the milk from the mother to the newborn, then boosting the growth of the same bacteria in the newborn’s intestine, influencing different functions such as the development of the gastrointestinal tract, the immune system, and the central nervous system of the baby [35]. However, it is noteworthy that although the “enteromammary pathway” process can establish a good microbiome environment, it also has the potential to move infectious agents such as immunodeficiency virus, herpes, and cytomegalovirus [36].

Breastfeeding shapes the development of the infant’s gut microbiota in the first years of life, both directly through the infant’s contact with the breast milk microbiota and indirectly through bioactive factors and compounds in breast milk that influence the growth and metabolism of bacteria [37]. Several studies have analyzed whether breast milk feeding practices (e.g., breastfeeding exclusivity, nursing at the breast, feeding pumped breastmilk from a bottle) may influence the transmission of bacteria from HBM to the newborn’s intestine [38,39]. For instance, the analysis conducted on 1249 mothers belonging to the CHILD (Canadian Healthy Infant Longitudinal Development) cohort study demonstrated that *Streptococcus* spp. and *Veillonella dispar* were simultaneously found in both HBM and baby’s feces and were reduced if babies received pumped HBM or if they began weaning [38]. Others [40] found that in *Bifidobacteria* (*Bifidobacterium bifidum* and *B*. *animalis*), most taxa shared between breast milk and newborn feces were found in 48% of direct breastfeeding compared to 30% of indirect breastfeeding. Again, the infant gut microbiota composition is strongly associated with the exclusivity and duration of breastfeeding [41]. Stewart et al., in the TEDDY (The Environmental Determinants of Diabetes in the Young) cohort study [42], showed that the gut microbiota of exclusively breastfed infants was related to a lower diversity but higher bacterial counts, and higher levels of *Lactobacillus* spp., *Bifidobacterium breve*, and *B. bifidum*, when compared to formula-fed infants; furthermore, the cessation of breastfeeding resulted in faster maturation of the gut microbiome, as marked by the *phylum Firmicutes* [42]. Fehr and colleagues [38] found increased co-occurrence of amplicon sequencing variant (ASV), both in HBM and infant stool, in still-breastfed babies at 12 months, suggesting that prolonged breastfeeding promoted continued transfer of bacteria via HBM. Cabrera R. and colleagues [7] also investigated HM composition in 18 women during different stages of lactation; the analysis revealed that *Weisella*, *Leuconostoc*, *Staphylococcus*, *Streptococcus*, and *Lactococcus* genera were predominant in colostrum samples, while in 1- and 6-months milk samples, *Veillonella*, *Leptotrichia*, and *Prevotella*, typical of the oral cavity, increased significantly [7].

## 3. Human Breast Milk Microbiota Is Influenced by Maternal Lifestyle Habits

Maternal lifestyle habits, such as diet, physical activity and exercise, sleep quality, smoking, alcohol consumption, and psychological distress (including stress, anxiety, or depression), during pregnancy and lactation can affect the health of both mother and child [43]. Currently, it has been established that the microbial diversity of HBM varies during the lactation stages, depending on many factors, such as genetics, health, antibiotic usage, demography, environmental differences, and mode of delivery [31].

Regarding lifestyle factors, maternal dietary habits [44,45,46,47,48,49,50,51], smoking habits [40], alcohol consumption [52], and psychological distress [53,54] were evaluated for their direct effect on the composition of the HBM microbiota; physical activity/exercise and sleep quality [55,56,57,58,59] were investigated for their influence on the maternal intestinal microbiota composition, hypothesizing their potential effect on the HBM microbiota through the enteromammary pathway. Nonetheless, even if the topic is still poorly investigated, a healthy lifestyle during pregnancy and breastfeeding is essential and should be recommended to ensure the shaping of an HBM microbiota that promotes the health and well-being of the newborn; the choices a mother makes regarding her diet, physical activity, stress management, and avoidance of harmful substances directly influence the microbial composition of her breast milk, which plays a critical role in the development of her child’s immune system, gut health, and overall long-term health.

### 3.1. Maternal Dietary Habits

Maternal dietary habits could play an important role in shaping the HBM microbiota by influencing microbiota diversity through changes in maternal gut microbiota diversity [44]; indeed, the mother’s gut and breast can undergo certain bacterial migration through the enteromammary pathway [45]. This could explain the origin of bacteria in milk that are not only found on the maternal skin or in the newborn’s mouth (e.g., contamination hypothesis), described as potential bacterial sources that shape the human milk microbiota [44]. Thanks to this evidence, intervention in the maternal diet during lactation may affect the composition of the maternal gut microbiota and, thus, regulate breast milk microbiota, as well as the intestinal microbiota of breastfed infants [45]. Studies [44,46,47,48,49,50,51] reported how different maternal dietary patterns, as well as single dietary nutrients, can influence the HBM microbiota shaping (Table 1).

For instance, considering the maternal diet as a whole, Marsh et al. [46] conducted a cross-sectional microbiome diversity analysis of 72 human milk samples to investigate the relationship between different maternal dietary patterns (e.g., vegan, vegetarian, and omnivore) and HBM microbiota composition [46]. No significant differences were observed between the diet groups at the phylum level [46]. However, statistically significant differences were observed between the vegan and omnivore groups at the genus level where of the 18 genera that were statistically different between the two groups, *Mycobacterium*, *Rothia*, *Geobacillus*, *Actinomyces*, and a genus of the family *Vermiphilaceae* were present in more than 30 samples, and differences in their relative abundance were significant after correction for false discovery rate [46]. Moreover, species indicator analysis (SIA) revealed several species to be significant positive indicators of omnivore, vegetarian, and vegan dietary patterns, and *Rothia mucilaginosa* was the most significant indicator associated with the omnivore group [46]. Similarly, Cortes-Macias and colleagues [47] conducted a cross-sectional study to investigate the influence of maternal diet and specific nutrients during pregnancy on HBM microbiota from healthy mothers (MAternal Microbes—MAMI, Spanish cohort), divided into the group with a high maternal intake of fiber, plant protein, and carbohydrates (Cluster I) and the group with a high maternal intake of animal protein and lipids (Cluster II) [47]. Contrary to what Marsh et al. [46] have reported, the authors highlighted that HBM microbiota was shaped by maternal dietary clusters with important contributions coming from dietary fiber and both plant and animal protein intakes [47]. Indeed, Cluster I showed (i) at the phylum level, a higher relative abundance of both *Bacteroidetes* and *Actinobacteria*; (ii) at the genus level, a higher relative abundance of *Staphylococcus* and *Lactobacillus*, *Bifidobacterium*, and *Sediminibacterium*; (iii) at the genus level, a lower relative abundances of *Bacteroides* and *Escherichia*/*Shigella*, when compared with Cluster II [47]. Moreover, LEfSe [linear discriminant analysis (LDA) > 2.5] analysis showed that Cluster II was characterized by *Bacteroides* and *Escherichia*/*Shigella* genera, whereas *Staphylococcus* spp. was characteristic of Cluster I [47]. Interestingly, the discrepancies between the findings highlighted by Marsh et al. [46] and Cortes-Macias et al. [47] may be due to the wide range of postpartum periods during which milk samples in the Marsh et al. [47] study were collected (2 weeks–over 2 years). It is known that the lactation phase (i.e., colostrum, transitional or mature milk) influences the microbiota of HBM, and although longitudinal studies characterizing human milk throughout the phase are lacking, that study showed that *Sphingobium* and *Pseudomonas* were enriched later during breastfeeding [60].

Regarding the contribution of maternal nutrient intake in shaping the HBM of lactating mothers, again, Marsh and colleagues in the same study previously described [46], classified HBM samples into low and high groups according to the content of fatty acids: saturated fats (SF, lower/greater than 40%), unsaturated fats (UF, lower/greater than 60%), and trans-unsaturated fatty acids (TF, lower/greater than 0.7%) [46]. The authors reported that TF breast milk content differed by dietary groups and highlighted the relationship between maternal diet and the microbial profile of human milk [46]. Indeed, the microbiota composition of HBM containing a greater content of TF differed significantly from that containing low TF; furthermore, differences in diversity between the high and low groups within both the SF and UF categories were noted, although these differences did not achieve statistical significance. [46]. SIA also identified 20 taxa as representative of the high UF group, including *Lactobacillus rhamnosus* and undetermined species of *Lactobacillus*; 21 taxa as representative of the high SF, including *Streptococcus* and *Bifidobacterium*, and 19 taxa as representative of the TF group, including *L. rhamnosus* and *Lactobacillus agilis* [46]. At the same time, Cortes-Macias et al. [47] reported an association between HBM microbiota and specific dietary nutrients; among these, it is noteworthy to mention that *Staphylococcus* and *Bifidobacterium* were associated with carbohydrate intake, whereas the *Streptococcus* genus was associated with intakes of the n–3 PUFAs (EPA and docosapentaenoic acid, 22:5ω-3).

Padilha and colleagues [44] explored in a cross-sectional study the effect of the maternal diet during pregnancy and the first month of lactation on the human milk microbiota profile of healthy lactating Brazilian women volunteers with uncomplicated pregnancies. The authors reported that the effects of maternal dietary habits appeared to be different for diet during pregnancy and the breastfeeding period, as diet during pregnancy had a greater impact on bacterial community structure than diet during the first month of breastfeeding, where specific nutrients, on the contrary, have been shown to influence the abundance of specific bacterial genera differentially [44]. Particularly, the vitamin C intake during pregnancy was positively correlated with the *Staphylococcus* genus, while PUFAs and linoleic fatty acid correlated positively with the relative abundance of the genera *Bifidobacterium* during the lactation period [44]. Moreover, *Pseudomonas* correlated negatively with sugars and positively with vitamin B_9_, while *Enterococcus* correlated negatively with vitamins B_1_, B_2_, and B_9_ [44].

Again, a recent cross-sectional study conducted by Londoño-Sierra and colleagues [48] on a group of women from Colombia during the first three months of lactation demonstrated that anthropometric indexes (e.g., body mass index, BMI), the gestational weight gain, and the macro and micronutrient intake during both gestation and lactation could impact the content of HBM bacterial genera, as well as the consumption of certain micronutrients, which could contribute to the presence of bacteria with probiotic potential [48]. Indeed, it has been observed that women with adequate BMI reported a higher alpha diversity even without statistical differences, while a lower richness and diversity were observed in those women who had excessive gestational weight gain. Regarding macronutrient intake, the consumption of simple carbohydrates during pregnancy and lactation also positively correlated with *Enterobacter* spp. and negatively correlated with the genus *Bifidobacterium* spp. during lactation [48]. Similarly, the maternal intake during lactation of total fat, saturated fat, and monounsaturated fat showed a positive correlation with the genus *Eubacterium* spp. [48]. Conversely, the maternal consumption during lactation of total protein, total carbohydrates, cholesterol, and dietary fiber negatively correlated with the genus *Aerococcus* spp. [48]. As for the micronutrient intake during lactation, a positive correlation was observed between (i) folic acid intake and *Akkermansia* spp.; (ii) B-group vitamins, including B_1_, B_2_, B_3_ and genus *Gemella* spp.; (iii) vitamin A intake and the genera *Bifidobacterium* spp., *Corynebacterium* spp. and *Ruminococcus UCG.009* spp. [48]. At the same time, the genus *Aerococcus* spp. negatively correlated with vitamin A, vitamin C, folic acid, pantothenic acid, and magnesium [48]. Significant associations were also observed between maternal macro and micronutrient intake during pregnancy and HBM microbiota composition [48]. Indeed, positive correlations were identified between the intake of simple and total carbohydrates, saturated fat, and total protein with the genus *Enterobacter* spp.; saturated fat intake also positively correlated with the genus *Halomonas* spp. [48]. Regarding micronutrient intake, zinc and vitamin C positively correlated with *Pseudomonas* spp. and *Rothia* spp., respectively [48]. On the contrary, protein and saturated fat intake were negatively correlated with *Bifidobacterium* spp., while fiber intake negatively correlated with *Ruminiclostridium 9* spp., *Ruminococcaceae UCG.005*, *Ruminococcaceae UCG.014*, and *Ruminococcus 1* spp. [48].

Others [49], in a prospective longitudinal study conducted on 47 HBM samples from mothers belonging to the Cambridge Baby Growth and Breastfeeding Study (CBGS-BF), have recently reported that adequate intake of non-digestible carbohydrates (e.g., fibers and resistant starch) could also promote the metabolism of the *Clostridiales*-dominant microbiota, resulting in the fermentation of short-chain Fatty Acids (SCFAs) in HBM, including butyrate, which is known to have anti-inflammatory properties and may be protective against increased adiposity in children [49,61]. Indeed, the authors observed cross-sectionally at 6 weeks of infant age that HBM butyrate content was associated with HBM microbiota composition [49]. However, the source of butyrate in HBM is still unclear. Indeed, some researchers consider that it is produced in the intestines and later transferred to breast milk through the enteromammary pathway [62]; others argue that it is a metabolite yielded by the microorganisms that colonize HBM, such as HMO [63,64].

It is important to highlight that the HBM samples in the above-mentioned studies all came from healthy mothers; however, there are also studies in the literature that consider mothers with gestational glucose intolerance [50] and celiac disease (CD) on a gluten-free diet [51]. Thus, LeMay-Nedjelski et al. conducted an explorative analysis to investigate the association between lactating diet and HBM microbiota at 3 months post-partum in 93 mothers with different degrees of gestational glucose intolerance. The results suggested that maternal consumption of fiber and fat were important determinants of the HBM microbiota [50]. Indeed, the authors demonstrated that polyunsaturated fat and fiber from grains were positively correlated with the *α*-diversity of HBM microbiota and negatively correlated with *Acinetobacter* incidence; trans fats showed a positive association with both *Staphylococcus* and *Gemella* incidence [50]. Additionally, fiber from grain intake was positively correlated with the *β*-diversity of HBM microbiota and associated with a reduced incidence of *Streptococcus* [50]. Again, Olshan and colleagues [51] cross-sectionally analyzed the HBM microbiome, with CD on a gluten-free diet and healthy controls ingesting gluten. The authors reported that the HBM composition of subjects with CD on a gluten-free diet appeared quite like the HBM composition of healthy control subjects at 7–14 days post-partum since they did not identify any significant differences in *α*- or *β*-diversity between groups at the species or strain levels [51]. However, milk samples of mothers adhering to a gluten-free diet resulted in an association with a significantly higher abundance of *Acinetobacter ursingii*, *Rothia mucilaginosa*, and *Acintobacter* spp. (*p* < 0.05) [51]; *Rothia mucilaginosa* has been detected in the gut microbiota of subjects with autoimmune inflammatory diseases [51].

### 3.2. Other Lifestyle Variables

#### 3.2.1. Maternal Physical Activity and Physical Exercise

During breastfeeding and lactation, regular physical activity (PA)/physical exercise (PE) may lead to several benefits for maternal health [65,66]; furthermore, moderate to vigorous PA does not impact the volume and quality of HBM if it is supported by adequate maternal nutrition and hydration only [65,66].

To our knowledge, no research has studied the effect of maternal PA or PE on the composition of the HBM microbiota; however, several studies have reported a positive impact of PA on the composition of the human gut microbiota, such as an increase in bacterial diversity and richness [55,56,57].

For instance, a recent systematic review was conducted by Cataldi et al. [56] to explore the current scientific evidence investigating the relationship between PA/PE and gut microbiota shaping, focusing on the different types/variables of PA/PE and age-related effects, both in healthy and unhealthy individuals. Results highlighted that gut microbiota diversity was associated with aerobic exercise contrary to resistance training; abundance of *Prevotella* genus was associated with training duration; exercising according to the minimum dose recommended by the World Health Organization (WHO) did not significantly change the gut microbiota richness and diversity; intense and prolonged PE can induce a higher abundance of pro-inflammatory bacteria [56]. Another systematic review [57] aimed at evaluating the role that PA played in determining the gut microbiota composition in healthy subjects (without gender or age limitations), trying to distinguish its effects from those of diet. In general, considering the results of the 10 studies included, the authors reported that PA can increase the abundance of health-promoting bacteria, hindering some negative genera [57]. Indeed, a higher variability and abundance of *Firmicutes* (genera *Ruminococcaceae* or *Fecalibacteria*) were reported in active subjects when compared to the inactive ones, especially in athletes; these findings were less robust in the studies performed in the general population because of the different volume of exercise between athletes and non-athletes, suggesting the influence of the PA volume in shaping the gut microbiota [57]. Bressa and colleagues [58] conducted an observational study on Caucasian childbearing-age women and compared the composition of the intestinal microbiota between women who did not practice any PE and women who at least practiced exercise at the minimum dose recommended by the WHO. Results showed PE did not produce significant changes to the microbiota diversity/richness [58]; however, sedentary parameters (i.e., sedentary time and breaks) correlated with microbiota richness (number of species and Shannon and Simpson indices), hypothesizing that physical exercise pattern, such as breaks in sedentary time, the avoidance of long periods of inactivity in daily life, might shape the intestinal microbiota [58]. In general, results reported that PA performed at low doses but continuously could increase the abundance of health-promoting bacteria, such as *Bifidobacterium* spp., *R. hominis*, *A. muciniphila*, and *F. prausnitzii* [58]. Indeed, although at the phylum level, a higher presence of *Firmicutes* and a lower presence of *Bacteroidetes* in active women with a trend toward significance was observed, at the genus level, there were significant differences in the following genera: *Bifidobacterium*; *Barnesiellaceae*; *Odoribacter*; *Paraprevotella*; *Turicibacter*; *Clostridiales*; *Coprococcus*; *Ruminococcus*; and two unknown genera of *Ruminococcaceae* family [58].

Therefore, based on the positive influence of PA in shaping gut microbiota and based on the fact that the maternal intestinal tract relates to two seemingly unrelated organs of the mammary gland, suggesting that bacteria in the mother’s gut migrate to the mammary gland via an endogenous pathway (active migration theory) [45], the modulation of the maternal gut microbiota through PA or PE could, albeit indirectly, influence the microbiota composition of the HBM [67].

#### 3.2.2. Quality of Sleep

Recently, a current study demonstrated that sleep quality was one of the predictors of breastfeeding self-efficacy since as women’s sleep quality increases, their self-efficacy in breastfeeding also increases [68]. Indeed, sleep deprivation (SD) is known to affect a mother’s quality of life and her mental health status [69,70], and it has been reported that continuous sleep shortage (four hours of sleep per day) lasting more than a month increased the risk of post-partum depression [71].

To date, several studies [59,72] have shown no association between sleep quality and HBM macro- and micronutrient composition; furthermore, to our knowledge, no study has evaluated the role of SD either on the microbial composition of HMB or on the composition of the maternal intestinal microbiota. However, Sun and colleagues [59], in a recent narrative review, depicted how SD causes gut microbiota dysbiosis, as well as systemic changes such as immune defense reduction, increased energy intake, broken glucose metabolism, and impaired cognitive functions. The results highlighted that SD might affect gut microbiota in both abundances and compositions, leading to a reduction in α-diversity and chao1 indexes [59]. Furthermore, SD results in (i) higher abundances of the families *Coriobacteriaceae* and *Erysipelotrichaceae*, (ii) lower abundance of the *Tenericutes* phylum, and reduction at the genus-level relative abundance of g_*Prevotella*; g_*Sutterella*, g_*Parasutterella*, g_*Alloprevotella*, g_*Anaeroplasma*, g_*Elusimicrobium*. Last, SD increased the ratio *Firmicutes:Bacteroidetes* [59]. Although the study examined in the review by Sun et al. [56] was conducted on a small sample, which sometimes involved only young, healthy men, the results seem promising and of interest for extending the investigation to larger populations, also including breastfeeding women. Indeed, ensuring good sleep hygiene and managing stress are, therefore, critical components of postnatal care where adequate quality of sleep is essential for breastfeeding mothers since sleep supports the balance of the mother’s gut microbiota, which directly affects the quality of HBM and, subsequently, the infant’s gut health and overall well-being.

#### 3.2.3. Tobacco Exposure

Tobacco exposure, either directly or indirectly, has harmful effects on human health and increases the risk of several diseases [73]. According to data, in the United States, 10.7% of women smoke during pregnancy, while in Europe, this habit involves 1 in 10 women [74]. Even if mothers stop smoking during pregnancy, 50–80% of them start again within six months after giving birth, during breastfeeding [74].

Evidence has highlighted the harmful effects of tobacco smoke, which contains over 5300 compounds and 70 carcinogens, as well as second-hand smoke exposure on the HBM composition, which can reduce the human milk protective properties and, thus, negatively impact infant health and development [75]. Indeed, breastfed infants of smoking mothers are more prone to experience adverse effects, including allergies, sleep disorders, increased colic, upper respiratory tract infections, cardiac rhythm disorders, and sudden infant death syndrome [75]; however, the mechanisms underlying the infant risks mentioned above, which are associated with exposure to smoking, are still under investigation. In addition, mothers who smoke and choose to breastfeed have a shorter lactation period and produce less milk compared to non-smoking mothers, particularly between weeks 2 and 4 after initiation of lactation [74]. These changes can be explained by the effects of nicotine on hormonal levels [74]. Studies have shown that nicotine in the mother’s bloodstream reduces prolactin levels; furthermore, maternal smoking habits during breastfeeding may alter the taste of milk, which may affect the baby’s desire to suck [74]. A recent systematic review concluded that milk produced by smoking mothers is associated with a lower macro- and micronutrient content [74], reduced antioxidant properties, and altered immune status [76,77]. Interestingly, mothers exposed to passive smoking showed higher levels of heavy metals such as arsenic, cadmium, mercury, and lead [75].

To our knowledge, only one cohort study by Moossavi et al. investigated the effect of smoking on the HBM microbiota and did not observe any effect on the diversity or composition of HBM. In this contest, researchers analyzed a subset of the CHILD cohort study, investigating the impact of several maternal and infant factors on the microbial composition of HBM [40]. They examined HBM samples produced 3–4 months after birth, considering both mothers who have always smoked and mothers who smoked during the prenatal period [40]. Smoking habit was associated with microbiota diversity in a phylum-specific manner; however, no association was highlighted between overall α-diversity in milk samples and smoking habit [40]. Thus, exposure to tobacco appears not to influence the composition of breast milk in microbial terms, although this relationship is poorly studied to date, and studies are needed to reach definitive conclusions. Similarly, the effect of cigarette smoking exposure on the gut microbiota composition remains unclear, and there is no agreement on how smoking affects the gut microorganisms.

However, others [78] compared the difference in gut microbial composition between current and never smokers in a Chinese cohort, showing that although there were no significant differences in the microbiota α-diversity among the two groups, smoking altered the relative abundance of several specific taxa, where *Phascolarctobacterium* and *Fusobacterium* increased, and *Dialister* decreased. Furthermore, this research revealed that smoking introduced more microbial interactions between taxa and a decrease in the network modularity [78]. Therefore, in this case, considering the maternal active migration theory previously elucidated [45], the habit of smoking during breastfeeding could somehow modify the composition of the HBM; this relationship deserves to be investigated more precisely.

#### 3.2.4. Alcohol Consumption

Alcohol consumption during pregnancy and lactation is generally not recommended due to the potential risks it poses to the developing fetus, such as Fetal Alcohol Spectrum Disorders (FASD), which can lead to physical, behavioral, as well as cognitive impairments, including low birth weight, facial abnormalities, learning disabilities, and behavioral problems [79], miscarriage and stillbirth [80], pre-term births, and births defects [80]. Moreover, alcohol passes into breast milk and can reach the baby’s bloodstream; the amount of alcohol in HBM generally peaks 30–60 min after consumption, but this time may be delayed if it is consumed with the meal [81]. Thus, also during lactation, alcohol exerts its harmful effects on the infant since even small amounts of alcohol in HBM can affect a baby’s sleep patterns, feeding, and development [82]; at the same time, excessive maternal alcohol consumption may interfere with the milk let-down reflex, potentially reducing milk production as well as lead to delays in infant motor development [82].

Since it has been established that alcohol consumption affects the composition of the gut microbiota, leading to dysbiosis and increased intestinal permeability [83,84], similar effects may be transferred on the microbial composition of the HBM either through the direct impact exerted by alcohol on the HBM or through the indirect effect through the enteromammary pathway [45], as described for the other factors; however, the literature is still scarce. For instance, a population-based birth cohort study conducted by Wang and colleagues [85] on mother–child dyads enrolled in central China investigated the effect of alcohol consumption and maternal diet during pregnancy on maternal and infant’s gut microbiota, showing that alcohol consumption during breastfeeding induced changes in the maternal gut microbiota shaping. Indeed, general linear model analysis performed to control possible confounders revealed that maternal alcohol consumption during pregnancy correlated negatively with *Faecalibacterium* while correlating positively with *Phascolarctobacterium* and *Blautia* [85]. In addition, although the α-diversity of maternal gut microbiota was not associated with alcohol consumption, the PCoA (β-diversity) revealed a significant difference in maternal gut microbiota composition between the alcohol-consumption group and the non-alcohol consumption group [85]. Again, Shenker and colleagues [52] evaluated cross-sectionally and, for the first time, the metabolite (rapid evaporative ionization mass spectrometry—REIMS—for metabolic fingerprinting) and bacterial composition of HBM from mothers of infants aged 3–48 months, also considering alcohol consumption, among other lifestyle factors. The authors showed that the macro-level metabolic and microbial composition of HBM was maintained between 3 and 24 months of nursing age. Following other previous studies, results reported a core genus-level microbiome within HBM, which was dominated by *Streptococcus* and *Rothia*, with other genera in lower-level abundance, including *Actinomyces*, *Acinetobacter*, *Veillonella*, *Granulicatella*, *Burkholderia*, *Sediminibacterium*, and *Corynebacterium* [52]. Lifestyle factors were correlated with microbiome features, but contrary to what was described by Wang and colleagues [82], no significant associations were described for alcohol consumption [52].

#### 3.2.5. Postnatal Psychosocial Distress

In breastfeeding women, postnatal psychosocial distress is a common issue that can lead to anxiety, stress, or depressive symptoms [53]. Although physiological mechanisms remained unclear, findings described a relation between maternal psychological distress (e.g., anxiety, perceived stress, depression) and non-optimal breastfeeding outcomes, including a decreased proportion and duration of exclusive breastfeeding [86].

A high level of maternal psychological distress may influence the HBM microbiota, reducing microbial diversity and altering the abundance of specific bacterial groups through both direct and indirect mechanisms [53,87]. Indeed, since stress can affect both gut microbial composition and intestinal permeability [88,89,90], the direct pathway could involve the transfer of aberrant maternal gastrointestinal microorganisms via the enteromammary pathway [53]; on the other hand, the indirect mechanism may impact the nutrient content in HBM, which could potentially affect the microorganism content in milk [53].

Browne and colleagues [53], in the longitudinal BINGO (Dutch acronym for Biological Influences on Baby’s Health and Development) cohort study, investigated for the first time the association between postnatal maternal stress and HBM microbial composition, analyzing milk samples at 2, 6, and 12 weeks after delivery. Variables related to stress, anxiety, and depressive symptoms were assessed, and subjects were grouped into women with high (H) and low (L) psychosocial distress [53]. Considering HBM microbial composition and psychological distress, no significant differences in the relative abundance of major bacterial genera were revealed [53]. However, progressive changes in the content of *Firmicutes*, *Proteobacteria*, and *Bacteroidetes* at the phylum level and *Acinetobacter*, *Flavobacterium*, and *Lactobacillus* at the genera level were found in the HBM of the L-women group [53]. Concerning milk microbial diversity, women with low psychosocial distress showed a significantly increased microbial α-diversity abundance [53].

Perinatal stress experienced by mothers of very premature infants can modulate HBM and the infant’s gut microbial composition [54]. Recently, a 2-year prospective study of mothers of very preterm newborns fed with mother’s milk was carried out, showing that maternal stress influenced bacterial diversity in the HBM as well as in fecal samples from their premature newborns with clear trends despite non-significant results (due to the study limitations) [54]. Indeed, the researchers described an inverse relationship between stress and HBM production in the first days of life and a progressive decrease and increase in the proportions of *Firmicutes* and *Proteobacteria* species, respectively, over 15 days post-delivery, in HBM of mothers with high stress levels [54]. The results of the studies investigating the influence of other lifestyle variables are summarized in Table 2.

## 4. Probiotics: Potential Bridge Between Breastfed and Formula-Fed Infants

Breastfed infants typically harbor a gut microbiota predominantly composed of *Bifidobacterium* and *Lactobacillus*, which are linked to beneficial immunological and metabolic effects [91]; these bacteria are selectively supported by HMOs, which act as prebiotics [91,92,93]. In contrast, formula-fed infants tend to exhibit a more diverse but less beneficial microbial profile, characterized by increased levels of potentially pathogenic bacteria, including *Clostridium difficile* and *Enterobacteriaceae* [94]. Studies demonstrated that these microbial imbalances in formula-fed infants have been associated with a heightened risk of infections, allergies, and metabolic disorders [95].

Probiotics, particularly strains of *Bifidobacterium* and *Lactobacillus*, have been explored for their ability to modulate the gut microbiota of formula-fed infants, potentially shifting it to resemble the microbiota of breastfed infants [96,97,98]. The premise is that introducing beneficial bacteria through probiotic supplementation may foster a more favorable gut environment akin to that induced by breastfeeding [97,98]. Studies showed that probiotics could increase the abundance of *Bifidobacterium* and *Lactobacillus* in formula-fed infants, promoting a microbial composition more similar to that of breastfed infants; however, the extent of this modulation depends on factors such as the probiotic strain, dosage, and duration of administration [99].

Currently, it has been reported [100,101] that probiotic supplementation in formula-fed infants has been associated with improved immune outcomes, including reductions in the incidence of diarrhea, necrotizing enterocolitis (NEC), and respiratory infections and suggesting that probiotics might help offset some of the immunological advantages conferred by breast milk. Similarly, from a metabolic perspective, probiotics may reduce inflammation and enhance gut barrier function, mimicking some of the protective effects of breast milk [102].

Nevertheless, there are limited long-term data on the sustained impact of probiotic-induced microbiota changes on the health outcomes of formula-fed infants, and several challenges and limitations must be acknowledged. In the first place, not all probiotics exert the same effect, and the beneficial outcomes observed with specific *Bifidobacterium* and *Lactobacillus* strains are not consistent across all strains, emphasizing the need for careful selection of probiotic species and strains to achieve the desired microbiota modulation [97].

Additionally, probiotics tend to provide only temporary colonization of the gut, and once supplementation ceases, the introduced strains may decline, and the infant’s microbiota may revert to its pre-supplementation state [97,99]. Therefore, continuous administration or long-term dietary changes may be necessary to maintain the benefits.

Last, probiotics alone, however, may not fully replicate the effects of HBM, particularly due to the absence of HMOs in most infant formulas. The inclusion of HMOs in the formula, along with probiotics, offers a more comprehensive strategy to emulate HBM microbiota-shaping effects since they serve as selective substrates for beneficial bacteria [91,103]. Studies indicate that formulas combining HMOs and probiotics, also named synbiotics, can produce a microbiota profile more similar to that of breastfed infants than probiotics alone [98]. However, further research is required to optimize synbiotic formulations and assess their long-term health impacts.

## 5. Discussion

The WHO recommends exclusive breastfeeding for the first six months, followed by continued breastfeeding alongside safe complementary foods until at least two years of age [104]. Breastfeeding offers numerous benefits, including improved infant health and development, strengthened maternal–infant bonding, and a lower risk of chronic conditions [105,106]. Human breast milk is increasingly recognized not only as a biological fluid supporting neonatal growth but also as a complex medium with functions that extend beyond its nutrient content [4,107]. The microbial composition of HBM is crucial for establishing the infant gut microbiota, and any alterations may pose health risks, with early dysbiosis linked to a higher risk of non-communicable diseases like obesity and type 2 diabetes in adulthood [108]. Maternal environmental factors, particularly lifestyle habits during pregnancy and lactation, significantly influence the microbiota of HBM [43,109]. Therefore, maintaining a healthy lifestyle—including proper nutrition, physical activity, quality sleep, and the avoidance of harmful behaviors—is essential for promoting immediate maternal and infant health and preventing long-term disease risk in offspring, potentially through the modulation of HBM components, especially its microbiota [13].

This narrative review summarizes for the first time the influence of these specific maternal lifestyle habits on the modulation of the HBM microbiota composition. Although other authors [110] have investigated the influence of maternal diet on HBM microbiota composition, the novelty of this review lies in the fact that, to date, maternal lifestyle as a whole has not been considered as a factor influencing microbial composition. This aspect is particularly significant as it aligns with the principles of Lifestyle Medicine, an evidence-based medical specialty that focuses on the prevention, treatment, and management of diseases through lifestyle changes [111]. It emphasizes the importance of making informed choices about diet, physical activity, sleep, stress management, and other lifestyle factors to improve overall health and well-being [111]. Indeed, lifestyle medicine is essential for promoting the health of both mothers and children by encouraging sustainable lifestyle changes that prevent disease and enhance well-being throughout the maternal–child relationship [112,113].

The scientific literature predominantly examines the role that the maternal diet (defined both as dietary patterns and as macro-and micronutrient intake) during pregnancy and breastfeeding has in modulating the microbial composition of HBM. Dietary patterns (e.g., vegan, vegetarian, and omnivore) may influence HBM microbiota composition at the species level [46], and the HBM of mothers with a high intake of plant-based proteins and complex carbohydrates have shown a different microbial composition of HBM [47,48] when compared to patterns characterized by a high content of simple sugars and animal proteins [47,48]. At the same time, the type of fats (SF, UF, and TF) significantly influenced the HBM microbiota uniformity [46]. To date, there are no studies analyzing the direct role that maternal PA/PE and quality of sleep during pregnancy and breastfeeding have in modulating the microbial composition of the HBM. However, because bacteria in the mother’s intestine migrate to the mammary gland via an endogenous pathway [45], the influence that reduced PA/PE and SD potentially exert on the maternal intestinal microbiota [57,58,59], although indirectly, could negatively modulate the composition of the HBM microbiota [45]. On the contrary, the positive influence of PE patterns and good sleep hygiene in increasing the abundance of beneficial bacteria for the health of the intestinal microbiota has been previously described [55,59]. Concerning the effects of cigarette smoking and alcohol consumption in the modulation of HBM composition, in the present narrative review, it was highlighted that, even only in two studies, a significant association has been reported between the consumption of these substances and the composition of the intestinal microbiota [78,85]; in contrast, no significant associations have been described regarding the direct effect of alcohol consumption on the composition of the HBM microbiota [52], as well as smoking habits and microbial diversity of the HBM [40].

Last, even if it is still poorly explored, researchers [53,54] reported how the reduction in maternal psychological distress progressively influenced the content of certain microorganisms both at the phylum at the genera level of the HBM, as well as the increase in its microbial α-diversity abundance, also in mothers of very premature infants.

Currently, probiotics can help bridge some microbiota differences between breastfed and formula-fed infants by promoting beneficial bacterial populations, particularly *Bifidobacterium* and *Lactobacillus* [96,97,99]. However, probiotics alone are unlikely to fully replicate the complex microbiota and immune-modulating effects conferred by HBM, especially in the absence of HMOs and other bioactive components of human milk [98]. A more holistic approach that combines probiotics with HMOs or other prebiotics may offer a more effective strategy for modulating the gut microbiota of formula-fed infants toward a breastfed-like profile [98].

It is, finally, important to mention that currently, bacterial analysis of HBM microbes, while essential for understanding the maternal–infant microbiome, faces several scientific and methodological limitations introducing heterogeneity in results and making it challenging to compare across studies. Sampling variability remains a major challenge; inconsistent collection methods (e.g., manual expression vs. pump extraction) can lead to contamination and variation in microbial load, while improper handling or delays in processing affect bacterial viability and diversity [30,114]. Additionally, contamination from breast skin and nipple microbiota complicates the distinction between native and external microbes, requiring sterile protocols that are not always rigorously applied [31,114].

The low-biomass nature of HBM further complicates analysis [114]. Indeed, compared to other biological fluids, HBM contains fewer bacterial cells, increasing the signal-to-noise ratio and making it difficult to distinguish native bacteria from environmental or reagent contaminants [30,31,114]. While common, techniques like 16S rRNA sequencing often struggle with sparse bacterial DNA, providing only taxonomic data at the genus level and lacking functional or strain-specific insights [27]. Whole-genome metagenomics, though more informative, is costlier and requires greater computational resources [115]. Data processing in bacterial studies adds another layer of complexity where high-throughput sequencing generates large datasets that require advanced bioinformatics tools, and factors such as sequencing errors, data normalization, and statistical models for low microbial abundance can influence results [115]. Moreover, most studies focus on bacterial identification, neglecting the functional roles of these microbes and techniques like metatranscriptomics, metabolomics, and proteomics are needed to assess microbial activity and interactions, but they are underutilized due to their complexity and expense [115]. The study design also presents limitations; for instance, cross-sectional studies, which analyze samples at a one-time point, offer limited insight into the dynamic nature of HBM microbiota [44,46,47,48,50,51,52,58,72]. On the contrary, longitudinal studies are more informative but are logistically demanding and costly [40,49,53,54,85]. Furthermore, associations between HBM microbiota and infant health outcomes, while frequently observed, remain correlative, as experimental studies that manipulate the microbiota to establish causality are ethically and practically challenging [30]. Finally, rare taxa with potentially significant roles often go undetected due to limitations in sequencing depth, with most methodologies focusing on more abundant species [30,114].

## 6. Conclusions

Human breast milk (HBM) is widely recognized as the optimal source of nutrition for infants, supporting both immediate growth and long-term health outcomes. This narrative review underscores the significant influence of maternal lifestyle habits—such as diet, physical activity, sleep quality, smoking, and alcohol consumption—on the composition of HBM microbiota. The findings suggest that maintaining a healthy lifestyle during pregnancy and lactation is essential not only for the mother’s well-being but also for shaping the infant’s gut microbiota and immune system development.

While the influence of diet on HBM microbiota is increasingly well-documented, other factors such as physical activity, sleep, and psychological stress remain underexplored. Current evidence indicates that lifestyle modifications during pregnancy and lactation could positively affect breast milk microbiota and, in turn, the infant’s health. However, more research, particularly longitudinal and interventional studies, is needed to fully understand the extent of these influences and to develop tailored recommendations.

Health professionals should integrate these findings into prenatal and postnatal care, advising mothers on the importance of maintaining a balanced diet, engaging in regular physical activity, and managing stress to promote optimal HBM composition. Additionally, public health policies must account for these factors when developing breastfeeding support programs, ensuring that maternal well-being is prioritized for the benefit of both mother and child.

A deeper understanding of how maternal lifestyle shapes HBM microbiota is crucial for designing effective interventions that promote infant health. Future research should focus on filling the gaps in our knowledge, particularly in areas like physical activity and stress, to develop comprehensive guidelines that will enhance breastfeeding practices and optimize long-term health outcomes for both mothers and their children.

At the same time, it is crucial to highlight that methodological and scientific limitations in studying the HBM microbiota, including inconsistencies in sampling, low microbial biomass, biases in detection technologies, and lack of functional insights, need to be addressed to gain more in-depth, accurate, and complete understanding. Standardizing protocols, using multi-omics approaches, and adopting longitudinal designs can help mitigate some of these limitations.

## Figures and Tables

**Table 1:** Studies evaluating the association between maternal dietary patterns and macro- and micro-nutrient intake during pregnancy and lactation and HBM microbiota composition.

Maternal Dietary Habits and HBM Microbiota Composition							
	Type of Dietary Pattern or Nutrients	Author(s) and Year	Type of the Study	Aim of the Study	HBM Samples Collection and Study Population	Microbiome Analysis Method	Results
Dietary pattern	Vegan Vegetarian Omnivore	Marsh et al., 2022 [46]	Observational and cross-sectional study	To investigate the relationship between different maternal dietary patterns (e.g., vegan, vegetarian, and omnivore) and HBM microbiota composition	A total of 72 HBM samples from healthy lactating mothers (mean age of the mothers: 32.1 ± 4.9 years; infants’ age range: from 3.5 to 186 weeks)	16S rRNA amplicon sequencing	(i) At the phylum level: no significant differences between the diet groups; (ii) At the genus level: significant differences in 18 genera between vegan and omnivore groups. *Mycobacterium*, *Rothia*, *Geobacillus*, *Actinomyces*, and a genus of the family *Vermiphilaceae* differed significantly (*p* < 0.001) in their relative abundance after correction for false discovery rate; (iii) At the species level: Species indicator analysis (SIA) revealed species to be significant positive indicators of omnivore (*n* = 9 species); vegetarian (*n* = 9 species); vegan (*n* = 12 species).
	High intake of plant protein, fiber, and carbohydrates (Cluster I) High intake of animal protein, lipids (Cluster II)	Cortès-Macias et al., 2021 [47]	Observational and cross-sectional study	To assess whether maternal diet and specific nutrients during pregnancy would shape the HBM microbiota	A total of 120 HBM samples (collected during 7–15 days after birth) from healthy lactating mothers (MAMI Spanish cohort)	16S rRNA amplicon sequencing	Cluster I showed (i) at the phylum level: higher relative abundance of *Bacteroidetes* (*p* < 0.001), *Actinobacteria* (*p* = 0.014); (ii) at the genus level: higher relative abundance of *Staphylococcus* (*p* = 0.036) and *Lactobacillus* (*p* = 0.022), *Bifidobacterium* (*p* = 0.026), *Sediminibacterium* (*p* < 0.001); a lower relative abundance of *Bacteroides* (*p* = 0.023) and *Escherichia*/*Shigella* (*p* = 0.023), when compared with Cluster II. LEfSe [linear discriminant analysis (LDA) > 2.5] analysis: - Cluster I was characterized by *Staphylococcus* spp.; - Cluster II was characterized by *Bacteroides* and *Escherichia*/*Shigella* genera.
	Gluten-free diet	Olshan et al., 2021 [51]	Case-control and cross-sectional study	To analyze the HBM microbiome integrated with metabolome profiling of subjects with CD on a gluten-free diet	A total of 36 HBM samples (collected at 7–14 days post-partum) from 20 mothers with CD on a gluten-free diet and 16 healthy mothers ingesting gluten	Multi-omics approach with shotgun metagenomics	No significant differences in *α*- or *β*-diversity between groups at the species or strain level (gluten-free diet vs. gluten ingestion); Significantly higher abundance of *Acinetobacter ursingii*, *Rothia mucilaginosa*, and *Acintobacter* sp. (*p*-value < 0.05) in women adhering to a gluten-free diet.
Macro and micronutrient intake	Fatty acids - SF, lower/greater than 40%; - UF, lower/greater than 60%; - TF, lower/greater than 0.7%	Marsh et al., 2022 [46]	Observational and cross-sectional study	To investigate the relationship between different maternal dietary patterns (e.g., vegan, vegetarian, and omnivore) and HBM microbiota composition	A total of 72 HBM samples from healthy lactating mothers (mean age of the mothers: 32.1 ± 4.9 years; infants’ age range: from 3.5–186 weeks)	16S rRNA amplicon sequencing	The microbiota composition of HBM containing a greater content of TF differed significantly from that containing low TF, Species indicator analysis (SIA) identified: - 20 taxa representative of the high UF group, including *Lactobacillus rhamnosus* and undetermined species of *Lactobacillus*; - 21 taxa representative of the high SF, including *Streptococcus* and *Bifidobacterium*; - 19 taxa representative of the TF group, including *L. rhamnosus* and *Lactobacillus agilis*.
	Carbohydrate n–3 PUFAs	Cortès-Macias et al., 2021 [47]	Observational and cross-sectional study	To assess whether maternal diet and specific nutrients during pregnancy would shape the HBM microbiota	A total of 120 HBM samples (collected during 7–15 days after birth) from healthy lactating mothers (MAMI Spanish cohort)	16S rRNA amplicon sequencing	Carbohydrate intake was associated with *Staphylococcus* and *Bifidobacterium;* n–3 PUFAs (EPA and docosapentaenoic acid, 22:5ω-3) intake was associated with the *Streptococcus* genus.
	Vitamin C (during pregnancy) PUFA/linoleic fatty acid (during lactation) Sugars Vitamin B group (e.g., B_1_, B_2_, B_9_) Lycopene (during pregnancy) Pectin (during pregnancy)	Padilha et al., 2019 [44]	Observational and cross-sectional study	To evaluate the effect of maternal diet during pregnancy, the first month of lactation on the HBM microbiota	A total of 94 HBM samples (collected at 30 ± 4) days after delivery) from healthy lactating Brazilian women volunteers with uncomplicated pregnancy	16S rRNA amplicon sequencing	The vitamin C intake was positively correlated (*p* = 0.029) with *Staphylococcus* genus and differed significantly between Cluster I (driven by *Streptococcus)* and Cluster II (driven by *Staphylococcus)* (*p* = 0.0249); PUFAs (*p* = 0.005) and linoleic fatty acid (*p* = 0.007) correlated positively with the relative abundance of the genera *Bifidobacterium*; *Pseudomonas* correlated negatively with sugars (*p* = 0.0085) and positively with vitamin B_9_ (*p* = 0.0053); *Enterococcus* correlated negatively with vitamin B_1_ (*p* = 0.0002), B_2_ (*p* = 0.0084), and B_9_ (*p* = 0.00001); Cluster 2 showed higher levels of pectin (*p* = 0.053) and lycopene (*p* = 0.058).
	Carbohydrates Total, monounsaturated, saturated fat Total protein Cholesterol Fibre B vitamin group (B_1_, B_2_, B_3_, B_9_) Vitamin A Vitamin C Pantothenic acid Magnesium	Londoño-Sierra et al., 2023 [48]	Observational and cross-sectional study	To analyze the effects of food and nutritional status during gestation and the first trimester of lactation on the microbiota of HBM	A total of 30 HBM samples from healthy women from Colombia in their first trimester of lactation	16S rRNA amplicon sequencing	Carbohydrate intake during pregnancy and lactation positively correlated with *Enterobacter* spp. (*p* ≤ 0.01 and *p* ≤ 0.01, respectively) and negatively with the genus *Bifidobacterium* spp. (*p* ≤ 0.01) during lactation; Total fat (*p* = 0.03), saturated fat (*p* = 0.03), and monounsaturated fat (*p* = 0.02) intake during lactation correlated positively with the genus *Eubacterium* spp.; Total protein (*p* = 0.01), total carbohydrates (*p* ≤ 0.01), cholesterol (*p* ≤ 0.01), and fiber (*p* ≤ 0. 01) negatively correlated with the genus *Aerococcus* spp. during lactation; Positive correlation for (i) folic acid intake and *Akkermansia* spp. (*p* ≤ 0.01) during lactation; (ii) B complex vitamins, including B_1_ (*p* = 0.01), B_2_ (*p* ≤ 0.01), B_3_ (*p* ≤ 0.01), and genus *Gemella* spp., during lactation; (iii) vitamin A, genera *Bifidobacterium* spp. (*p* = 0.047), *Corynebacterium* spp. (*p* = 0.01), and *Ruminococcus UCG.009* spp. (*p* ≤ 0.01) during lactation; Negative correlation between genus *Aerococcus* spp. and vitamin A (*p* = 0.01), vitamin C (*p* ≤ 0.01), folic acid (*p* ≤ 0.01), pantothenic acid (*p* ≤ 0.01), and magnesium (*p* ≤ 0.01) during lactation; Positive correlations between the intake of simple (*p* ≤ 0.01) and total carbohydrates (*p* = 0.02), saturated fat (*p* = 0.03), and total protein (*p* = 0.04) with the genus *Enterobacter* spp. during pregnancy; Saturated fat also positively correlated with the genus *Halomonas* spp. (*p* = 0.02) during pregnancy; Zinc and vitamin C positively correlated with *Pseudomonas* spp. (*p* = 0.03) and *Rothia* spp. (*p* = 0.01) during pregnancy, respectively; Protein (*p* = 0.01) and saturated fat (*p* ≤ 0.01) were negatively correlated with *Bifidobacterium* spp. during pregnancy; Fibre negatively correlated with *Ruminiclostridium 9* spp. (*p* = 0.02), *Ruminococcaceae UCG.005* (*p* = 0.03), *Ruminococcaceae UCG.014* (*p* = 0.02), and *Ruminococcus 1* spp. (*p* ≤ 0.01) during pregnancy.
	Non-digestible carbohydrates (e.g., fibers and resistant starch)	Olga et al., 2023 [49]	Prospective longitudinal study (Cambridge Baby Growth and Breastfeeding study, CBGS-BF)	To provide evidence on the origin of HBM butyrate by examining its associations with HBM microbiota composition	A total of 47 HBM samples (collected at 6 weeks infant age) from healthy mothers of the CBGS-BF study	16S rRNA amplicon sequencing	Non-digestible carbohydrates promoted the metabolism of the *Clostridiales-*dominant microbiota, resulting in the fermentation of SCFAs (e.g., butyrate); HBM butyrate content was associated with HBM microbiota composition (*p* = 0.036).
	Fiber from grains Fat (Polyunsaturated and Trans)	LeMay-Nedjelski et al., 2021 [50]	Observational and cross-sectional study	To assess the association between lactating diet and HBM microbiota at 3 months post-partum in mothers with gestational glucose intolerance	A total of 93 HBM samples (collected at 3 months post-partum) from mothers with different degrees of gestational glucose intolerance	16S rRNA amplicon sequencing	Polyunsaturated fats (*p* = 0.047) and fiber from grains (*p* = 0.048) positively correlated with α-diversity (Shannon Index) and negatively correlated with *Acinetobacter* incidence; Fiber from grains was positively correlated (*p* = 0.04) with *β*-diversity (Bray–Curtis dissimilarity index) and associated with a reduced incidence of Streptococcus; Trans fats showed a positive association with both S*taphylococcus* and *Gemella* incidence.

Legend. HBM: human breast milk; SF: saturated fatty acids; UF: unsaturated fatty acids; TF: trans-unsaturated fatty acids; PUFAs: polyunsaturated fatty acids; SCFAs: short-chain fatty acids.

**Table 2:** Studies evaluating the association between lifestyle factors (e.g., physical activity/physical exercise, quality of sleep, smoking habit, postnatal psychosocial distress) and gut or HBM microbiota composition.

	Author(s) and Year	Type of the Study	Aim of the Study	Samples Collection and Study Population	Microbiome Analysis Method	Results
Regular physical activity (PA)/physical exercise (PE)	Cataldi et al., 2022 [56]	Systematic review	To explore the current scientific evidence investigating the relationship between PA/PE and gut microbiota shaping, both in healthy and unhealthy general population	A total of 25 studies (randomized and non-randomized trials, observational studies) conducted on healthy and unhealthy subjects (no age restrictions, both sexes), following: aerobic PA/PE (*n* = 11); changes occurring after an endurance protocol (*n* = 2); changes induced by concurrent training (*n* = 3) or separate interventions of resistance and aerobic exercises (*n* = 3); effects produced from the practice of specific sports (*n* = 6).	16S rRNA amplicon sequencing; Metagenomic whole-genome shotgun sequencing.	Gut microbiota diversity is associated with aerobic exercise contrary to resistance training; Abundance of *Prevotella* genus was associated with training duration; Exercising according to the minimum dose recommended by the WHO did not change significantly the gut microbiota richness and diversity; Intense and prolonged PE can induce a higher abundance of pro-inflammatory bacteria.
	Dorelli et al., 2021 [57]	Systematic review	To investigate the role that PA plays in determining the gut microbiota composition in healthy humans, trying to distinguish its effects from those of diet.	A total of 10 studies (any study design with a control group) conducted on athletes (*n* = 5), active people classified based on habitual PA level (*n* = 3), and sedentary subjects undergoing exercise interventions (*n* = 2), without gender or age limitations.	16S rRNA amplicon sequencing	PA increases the abundance of health-promoting bacteria, hindering some negative genera; Higher variability and abundance of *Firmicutes* (genera *Ruminococcaceae* or *Fecalibacteria*) in athletes than sedentary people; Influence of the PA volume in shaping the gut microbiota.
	Bressa et al., 2017 [58]	Cross-sectional and case-control study	To compare the composition of the gut microbiota between individuals who did not practice any physical exercise and those who, at the very least, practiced exercise at the minimum dose recommended by WHO.	A total of 40 stool samples from Caucasian women (aged: 18–40; exclusion criteria; any kind of pathology, previous gastrointestinal surgery, antibiotics intake during three months before this study, smoking, prebiotics, probiotics, vegetarian or vegan, nutritional or ergogenic complements, pregnancy or lactation) divided into women who did not practice any PE (*n* = 21) and women who at least practiced exercise at the minimum dose recommended by the WHO (*n* = 19).	16S rRNA amplicon sequencing	PE did not produce significant changes to the microbiota diversity/richness; Sedentary parameters (i.e., sedentary time and breaks) correlated with microbiota richness (number of species and Shannon and Simpson indices); At the phylum level, a higher presence of *Firmicutes* (*p* = 0.085) and a lower presence of *Bacteroidetes* (*p* = 0.076) in active women, with a trend towards significance; At the genus level, significant abundance differences (*p* < 0.001) in the following: *Bifidobacterium* (↑active)*; Barnesiellaceae* (↑sedentary)*; Odoribacter* (↑sedentary)*; Paraprevotella* (↑active)*; Turicibacter* (↑active)*; Clostridiales* (↑active)*; Coprococcus* (↑active)*; Ruminococcus* (↑sedentary); and two unknown genera of *Ruminococcaceae* family (↑active); Significant higher abundance of healthy bacteria such as *F. prautznnii* (*p =* 0.029), *R. hominis* (*p* = 0.005), and *A. muciniphila* (*p* = 0.002) in active than in sedentary women.
Quality of sleep	Sun et al., 2023 [59]	Narrative review, including 4 representative studies conducted on animal models (mice, *n* = 2) and human population (*n* = 2; one randomized within-subject crossover study and one longitudinal clinical trial.	To summarize the gut microbiota dysbiosis caused by SD and the related diseases, elucidating the potential biological mechanisms.	A total of 25 stool samples from healthy participants (13M/12F) enrolled in the clinical trial; 9 healthy young men (age 23.3 ± 0.6 years) enrolled in the randomized within-subject crossover study.	16S rRNA amplicon sequencing	Only results from studies on humans were considered in the present review. SD may reduce dramatically diversity, including α-diversity (*p* < 0.001) and chao1 indexes (*p* = 0.011); SD may affect gut’s microbiota in abundance and compositions, leading to a reduction in *Bacteroidetes* and an increase in the ratio *Firmicutes:Bacteroidetes*; SD results in higher abundances of the families *Coriobacteriaceae* and *Erysipelotrichaceae* (*p* = 0.049); SD results in lower abundance of *Tenericutes* (*p* = 0.03); SD results in reduction in genus-level relative abundance (e.g., *g_Prevotella*, *p* < 0.001*; g_Sutterella*, *p* = 0.047; *g_Parasutterella*, *p* = 0.030; *g_Alloprevotella*, *p* < 0.001; *g_Anaeroplasma*, *p* = 0.001; *g_Elusimicrobium*, *p* = 0.001).
Smoking habits	Moossavi et al., 2019 [40]	Cohort study	To profile the HBM microbiota and to examine the association of maternal, infant, early-life, and milk factors with HBM microbiota composition	A total of 393 HBM samples (collected at 3–4 months postpartum) from healthy mother–infant dyads.	16S rRNA amplicon sequencing	Smoking habit was associated with microbiota diversity in a phylum-specific manner; No association was highlighted between overall α-diversity in HBM samples and smoking habits.
	Zhu et al., 2024 [78]	Observational, cross-sectional, and case-control study	To compare the difference in gut microbial composition between current and never smokers in the Chinese cohort.	A total of 80 stool samples from participants grouped as current smokers (*n* = 33, with 25 years of smoking experience, on average, and consumption of an average of 15 cigarettes per day) or never smokers (*n* = 47).	16S rRNA amplicon sequencing	No significant differences in the diversity (e.g., ASV number and Chao1, Shannon, and Simpson indices) of the gut microbiota between the current and non-smokers; Smoking altered the relative abundance of several specific taxa, where *Phascolarctobacterium* and *Fusobacterium* increased and *Dialister* decreased.
Alcohol consumption	Wang et al., 2021 [85]	Population-based birth cohort study	To analyze the effect of maternal alcohol consumption and diet during pregnancy on maternal and infant gut microbiota composition	A total of 29 stool samples both from mother and their infants enrolled in central China. Women were divided into the alcohol consumption group (*n* = 10) and the non-alcohol consumption (*n* = 19) group.	16S rRNA amplicon sequencing	Maternal alcohol consumption during pregnancy correlated negatively with *Faecalibacterium* (*p* = 0.001), while positively with *Phascolarctobacterium* (*p* = 0.032), and *Blautia* (*p* = 0.019) (GLM analysis); α-diversity of maternal gut microbiota was not associated with alcohol consumption; PCoA (β-diversity) revealed a significant difference in maternal gut microbiota composition between the alcohol-consumption and the non-alcohol-consumption groups (*p* = 0.006).
	Shenker et al., 2020 [52]	Cross-sectional study	To investigate the metabolite and bacterial composition of HBM, also considering alcohol consumption, among other lifestyle factors.	A total of 62 HBM samples (collected at infants’ ages of 3–48 months). Sample collection was carried out as a sub-project of the Breastmilk Epigenetic Cohort Study (Imperial College Healthcare Tissue Bank). Only 46 samples were suitable for the microbic profile analysis.	16S rRNA amplicon sequencing	Identification of a core genus-level microbiome within HBM, dominated by Streptococcus and Rothia, with other genera in lower-level abundance, including *Actinomyces*, *Acinetobacter*, *Veillonella*, *Granulicatella*, *Burkholderia*, *Sediminibacterium*, and *Corynebacterium* (REF); No significant association between microbial composition and alcohol consumption
Psychological distress	Browne et al., 2019 [53]	Longitudinal BINGO cohort study	To explore the association between postnatal maternal stress and HBM microbial composition	A total of 77 HBM samples milk samples (*n* = 51 with complete data) (collected at 2, 6, and 12 weeks after delivery) from women grouped into subjects with high (H, *n* = 13) and low (L, *n* = 13) psychosocial distress.	16S rRNA amplicon sequencing	No significant differences in the relative abundance of major bacterial genera were revealed; L-women group showed a significantly increased (*p* = 0.004) microbial α-diversity abundance (Shannon and Simpson index); Progressive changes in the content of *Firmicutes*, *Proteobacteria*, and *Bacteroidetes* at the phylum level and *Acinetobacter*, *Flavobacterium*, and *Lactobacillus* at the genera level were found in the HBM of the L-women group.
	Fernández-Tuñas et al., 2023 [54]	2-year prospective study	To investigate the association between postnatal maternal psychological distress and HBM production and microbial composition through the process of bacterial colonization of the gut of very preterm infants	A total of 45 HBM samples (collected on days 3, 7, and 15 after the birth) from mothers of very preterm newborns (*n* = 52; GA ≤ 32 weeks and/or bodyweight ≤ 1500 g) and 52 infant stool samples.	16S rRNA amplicon sequencing	Inverse relationship between stress and HBM production in the first days of life (*p* = 0.012); Progressive decrease and increase in the proportions of *Firmicutes* and *Proteobacteria* species, respectively, over 15 days post-delivery in HBM of mothers with high-stress levels.

Legend. HBM: human breast milk; WHO: World Health Organization; GLM: general linear model analysis.

## Data Availability

No new data were created or analyzed in this study. Data sharing is not applicable to this article.
